# Hospital‐treated bipolar disorder in adolescence in Finland 1980–2010: Rehospitalizations, diagnostic stability, and mortality

**DOI:** 10.1111/bdi.13486

**Published:** 2024-08-12

**Authors:** Anna Repo, Riittakerttu Kaltiala, Timo Holttinen

**Affiliations:** ^1^ Faculty of Medicine and Health Technologies Tampere University Tampere Finland; ^2^ Department of Adolescent Psychiatry Tampere University Hospital Tampere Finland; ^3^ Vanha Vaasa Hospital Vaasa Finland

**Keywords:** adolescent psychiatry, bipolar disorder, diagnostics, inpatient treatment, mental disorder, register study

## Abstract

**Aims:**

Estimates of the occurrence of bipolar disorder among adolescents vary from country to country and from time to time. Long delays from first symptoms to diagnosis of bipolar disorder have been suggested. Studies among adults suggest increased mortality, particularly due to suicide and cardiovascular diseases. We set out to study the prognosis of adolescent onset bipolar disorder in terms of rehospitalizations, diagnostic stability, and mortality.

**Methods:**

The study comprised a register‐based follow‐up of all adolescents admitted to psychiatric inpatient care for the first time in their lives at age 13–17 during the period 1980–2010. They were followed up in the National Care Register for Health Care and Causes of death registers until 31 December 2014.

**Results:**

Incidence of bipolar disorder among 13‐ to 17‐year‐old adolescents over the whole study period was 2.8 per 100, 000 same aged adolescents, and across decades, the incidence increased six‐fold. Patients with bipolar disorder during their first‐ever inpatient treatment were rehospitalized more often than those treated for other reasons. Conversion from bipolar disorder to other diagnoses was far more common than the opposite. Mortality did not differ between those firstdiagnosed with bipolar disorder and those treated for other reasons.

**Conclusion:**

The incidence of adolescent onset bipolar disorder has increased across decades. The present study does not call for attention to delayed diagnosis of bipolar disorder. Adolescent onset bipolar disorders are severe disorders that often require rehospitalization, but diagnostic stability is modest. Mortality is comparable to that in other equally serious disorders.

## 1 INTRODUCTION

Bipolar disorder is a mood disorder characterized by alternation between manic, depressive, and mixed episodes. Type I bipolar disorder includes all kinds of episodes (manic, depressive, and mixed), and type II is characterized by depressive and hypomanic episodes. Of affected individuals, 30% attempt suicide during their lifetime.^1^ Bipolar disorder usually begins during adolescence or in early adulthood, the peak incidence being at ages 15–24.^2^

The clinical picture of bipolar disorder among children and adolescents complicates diagnostic process, as illustrated in commonly first emerging diagnoses of ADHD, depression, and schizophrenia. Delayed diagnosis may complicate recovery.^3^, ^4^, ^5^ Early‐onset bipolar disorder may influence the fundamental developmental tasks of adolescence and have a negative impact on the self‐image of a juvenile.^5^ Earlier access to health care is important because young age at onset predicts a more severe clinical picture of bipolar disorder.^6^

Research on the incidence and prevalence of bipolar disorder has often been carried out among mixed samples of adolescents and adults or adolescents and children, leaving unclear the picture of epidemiology at different developmental stages. The 12‐month prevalence for bipolar disorder subtypes I and II among adults in Europe is about 1% in general population.^7^ In the Finnish population, the annual rate of all hospitalized patients (15 years old or older) with bipolar disorder in 1994 was 0.03%, of whom only 2% were under 20 years. The incidence of first‐episode admission for bipolar disorder among 10‐ to 19‐year‐olds was 1.7/100,000.^8^ This was a low incidence compared to the corresponding estimates in other parts of Europe and in the USA.^9^ A recurring trend in earlier studies has also shown that the incidence of inpatient‐treated bipolar disorder has increased significantly among adolescents and adults in the 21st century.^10^, ^11^, ^12^ Low prevalence of hospitalized patients and low incidence of inpatient‐treated bipolar disorder among adolescents in Finland may be due to late‐onset age or to patients not being hospitalized and/or treated only as outpatients.^8^, ^9^, ^13^ No new studies on bipolar disorder among adolescents in Finland have been published recently. The estimates of bipolar disorder among adolescents vary from country to country and from time to time, leaving ample need for further study; according to a meta‐analysis, the variation is to be considered due to applying nonstandard diagnostic criteria and unsystematic focusing on either narrow definitions of PBD or the full spectrum.^14^

Among adults it may take 5–10 years from the onset of symptoms before a patient suffering from bipolar disorder receives this diagnosis^15^, ^16^, ^17^, ^18^ and duration of untreated illness is longer in earlier onset cases, likely due to their more complex presentations.^16^ The clinical picture of pediatric and adolescent bipolar disorder is reminiscent of those of other disorders. Symptoms common to both bipolar disorder and ADHD are hyperactivity, inability to concentrate, impulsivity, and restlessness.^4^ Adolescent mania and schizophrenia share similar symptoms, such as various types of hallucinations, incoherence, and delusions.^5^ Mood‐congruent delusions, such as grandiosity, are typical of mania. Psychotic symptoms are in line with patient's mood in bipolar disorder, whereas delusions in schizophrenia are independent of patient's mood.^19^ A late diagnosis likely hampers adolescents' recovery and may cause treatment with suboptimal medication.^3^, ^5^, ^9^, ^15^ There may be variation in clinical practice regarding the diagnostic delay and the circumstances under which a prior diagnosis is later converted to bipolar disorder. It is not known to what extent a diagnosis of bipolar disorder later converts to other diagnoses.

According to a systematic review from 2021 including 42 publications,^6^ the recovery rate from an index episode of early‐onset bipolar disorder was 81.5%–100% and the risk of recurrence 35%–67%. The rehospitalization rate in bipolar disorder is not known. Research suggests that the diagnosis of a juvenile mainly changes between bipolar disorder subtypes. However, in some cases, a bipolar disorder diagnosis may later turn into a schizophrenia diagnosis.^6^ Studies among adults suggest increased mortality, particularly due to suicide and cardiovascular diseases.^20^, ^21^, ^22^, ^23^, ^24^, ^25^, ^26^, ^27^ Given the notable prevalence of early‐onset bipolar disorder among adults diagnosed with the condition, it is probable that adolescent onset bipolar disorder is associated with excess mortality.^28^, ^29^, ^30^, ^31^

Finland has a unique developmental stage‐specific adolescent psychiatric service system.^32^ This can be expected to improve diagnostic accuracy during the developmental phase and developmentally appropriate treatment may further improve prognosis. The National Finnish Care Register for Health Care^33^ provides an exceptional chance to examine morbidity in the population over decades. Therefore, we set out to explore the incidence of hospital‐treated adolescent bipolar disorder from 1980 to 2010, rehospitalizations, and stability of the diagnosis of bipolar disorder. In addition, we explored the incidence of bipolar disorder after hospitalization due to another disorder in adolescence, and which disorders first diagnosed in adolescence are at the highest risk of later changing to bipolar disorder. Finally, we compared mortality in bipolar disorder diagnosed in adolescence to that in other inpatient‐treated psychiatric disorders.

## 2 MATERIALS AND METHODS

The study comprises a register‐based follow‐up of all adolescents admitted to psychiatric inpatient care for the first time in their lives at age 13–17 during the period 1980–2010. All individuals fulfilling these criteria were identified in the National Care Register for Health Care (CRHC) and followed up until 31 December 2014, or until death, which was extracted from the national Causes of Death register. Reporting inpatient treatments to CRHC is mandatory. The patient's identity code including date of birth and sex, dates of hospital admission and discharge, diagnoses, treating institution, and medical specialty are recorded for all inpatient periods in any health care institutions.

First, incidence of bipolar disorder diagnosis (ICD‐10 F30 or F31) in the study population in first inpatient care was investigated. Percentages of all patients admitted and cumulative incidence per 100,000 same aged population were calculated. Cross‐tabulations with chi square statistics were used to investigate the incidence of bipolar disorder and differences of incidence between sexes, age at first inpatient care (categorized to early (13–14 years) and middle (15–17 years) adolescence) and decade of index admission (1980–89, 1990–99, 2000–2010). For the incidence of bipolar disorder diagnosis in follow‐up for those with no bipolar diagnosis during their first inpatient care period information on subsequent inpatient care was obtained. Subsequent incidence rates were calculated per 100 follow‐up years. Follow‐up time for each person was counted from discharge from the first admission to the diagnosis of bipolar disorder, death of the subject or end of 2014, whichever came first. For predictors for later bipolar diagnosis cross‐tabulations with chi square statistics were used to investigate associations of sex, categorized year of index admission, and main diagnosis during first inpatient care.

The stability of bipolar diagnosis in the study population was investigated in those for whom a bipolar disorder diagnosis was set during their first inpatient care. For this information on last inpatient care was obtained. Cross‐tabulations with chi square statistics were used to investigate the stability of diagnoses and associations of sex and age at first inpatient care and decade of index admission.

Cox regression analysis was used to assess hazard ratios with 95% confidence intervals for first bipolar disorder diagnosis recorded as the reason for inpatient treatment after index admission. For Cox regression analysis, follow‐up time was calculated from the discharge day of the index inpatient care period to the admission day to the first inpatient care with bipolar disorder diagnosis, date of death or last possible follow‐up day (31 December 2014) whichever was first. Bipolar disorder diagnosis in inpatient care was defined as an event. Sex, categorized admission year of first inpatient care period, and main diagnosis group at index admission were used as covariates.

Crude mortality and mortality rate per 1000 follow‐up years were calculated for those with and without a diagnosis of bipolar disorder at the index admission.

## 3 RESULTS

### 3.1 How common was bipolar disorder diagnosis during the first‐ever inpatient treatment period?

Bipolar disorder (F30 or F31) was the primary or secondary diagnosis of 286/17112 patients (1.7%) during their first‐ever hospitalization. The percentage of adolescents with bipolar disorder diagnosis fluctuated between the decades, but the absolute number of cases increased among both boys and girls across decades. Treated incidence per 100,000 over the whole study period was 2.8, increasing sixfold from the 1980s to the first decade of the 2000s. (Table 1) During the first‐ever inpatient treatment period 22% (63/223) of those with bipolar disorder diagnosis had the diagnosis of manic episode (F31.0, F31.1 or F31.2), whereas 78% (223/286) had another F31‐diagnosis.

**TABLE 1:** Bipolar disorder as the main or secondary diagnosis in adolescence (13–17 years) in different decades among first‐time psychiatric patients (% of admissions, *n*/*N*, cumulative incidence per 100,000 adolescents aged 13–17).

	1980–2010	1980–1989	1990–1999	2000–2010	*p* (between decades)
All					
% of admissions	1.7	1.0	2.5	1.6	<0.001
*n*/*N*	286/17,112	26/2588	90/3602	170/10,922	
Incidence per 100,000	2.8	0.8	2.8	4.8	
Males					
% of admissions	1.3	0.6	2.0	1.3	0.005
*n*/*N*	91/6873	9/1401	32/1594	50/3828	
Incidence per 100,000	1.8	0.5	1.9	2.7	
Females					
% of admissions	1.9	1.4	2.9	1.7	0.001
*n*/*N*	195/10,239	17/1187	58/2008	120/7044	
Incidence per 100,000	3.9	1.0	3.6	6.9	
*p* (male vs. female)	0.004	0.05	0.09	0.09	

Bipolar disorder was throughout the study period more common among girls (Table 1). The proportion of bipolar diagnoses during first‐ever psychiatric hospitalization was greater in the group of middle adolescents (aged 15–17) than that of early adolescents (aged 14–14), both among boys and girls (boys: 1.7% vs. 0.6%, *p* < 0.001; girls: 2.5% vs. 0.6%, *p* < 0.001).

### 3.2 Rehospitalization

Of those diagnosed with bipolar disorder during their first‐ever hospital stay, 67.1% had subsequent periods of treatment, whereas of adolescents whose main reason for first‐ever hospitalization was due to other diagnoses, 57.1% ended up being rehospitalized (*p* < 0.001). Across decades, the proportion of rehospitalized adolescents decreased both in the bipolar disorder group and among those treated due to other diagnoses (Table 2).

**TABLE 2:** Proportion of adolescents with and without a diagnosis of bipolar disorder rehospitalized in different decades (%).

	Bipolar disorder during the first period of treatment		*p*
	Yes	No	
1980–1989	84.6 (4/26)	71.6 (1835/2562)	0.143
1990–1999	66.7 (60/90)	65.7 (2308/3512)	0.851
2000–2010	64.7 (110/170)	51.0 (5481/10,753)	<0.001
1980–2010	67.1 (192/286)	57.2 (9624/16,826)	0.001

### 3.3 Diagnostic stability of bipolar disorder

Of those adolescents with bipolar diagnosis on their first‐ever period of inpatient treatment (*N* = 192), 41.7% (80/192) retained their diagnosis until their most recent hospitalization, with boys equally often as girls (44.4% (28/63) vs. 40.3% (52/129), *p* = 0.585). The diagnosis was retained equally often among those first diagnosed in early adolescence and those diagnosed in middle adolescence (36.4% (8/22) vs. 42.2% (72/170), *p* = 0.592). Bipolar diagnosis from 1980 to 1989 was retained in latest admission among 40.9% (9/22), that from 1990 to 99 in 23.3% (14/60), and bipolar diagnosis from 2000 to 2010 in 51.8% (57/110) (*p* = 0.002).

Most (43.8%) of the bipolar disorder diagnoses that were changed to another diagnosis between the first‐ever and most recent period of inpatient treatment fell into the schizophrenia group (F20‐29), in the latest treatment period. Of the diagnoses that changed, 17.0% ended up as other mood disorders (F32‐39). The third largest share (12.5%) of the changed diagnoses were later specified as personality disorders. No difference was found between boys and girls in the conversion of the diagnoses.

Among the patients with a diagnosis of a bipolar disorder on their index hospitalization, the diagnosis of bipolar disorder was retained on their latest inpatient treatment more commonly if the index hospitalization was for a manic episode (F31.0, F31.1 or F31.2) than if it was for another F31‐diagnosis (68% (34/50) vs. 32% (46/142), *p* < 0.001).

### 3.4 Subsequent conversion to bipolar disorder

In a later treatment period, 8.2% of those diagnosed with other than bipolar disorder at index admission received a diagnosis of bipolar disorder (Table 3). The absolute number and incidence rate per 100 person‐years of subsequent diagnosis of bipolar disorder increased across the decades. All indices of conversion to bipolar disorder were higher among females than males (Table 3).

**TABLE 3:** Bipolar disorder (F30 or F31) as primary or secondary diagnosis in a later treatment period among those with a different diagnosis during the first‐ever treatment period (%, *n*/*N*, Incidence Rate per 100 follow‐up years).

	1980–2010	1980–1989	1990–1999	2000–2010	*p* (between decades)
All					
% of admissions	8.2%	10.2%	9.4%	7.0%	<0.001
*n*/*N*	787/9624	188/1835	217/2308	382/5481	
IR	0.60	0.40	0.54	0.87	
Males					
% of admissions	6.5%	8.7%	6.3%	5.5%	0.004
*n*/*N*	258/3977	87/1004	68/1085	103/1888	
IR	0.43	0.35	0.35	0.67	
Females					
% of admissions	9.4%	12.2%	12.2%	7.8%	<0.001
*n*/*N*	529/5647	101/831	149/1223	279/3593	
IR	0.74	0.47	0.70	0.98	
*p* (male vs. female)	<0.001	0.014	<0.001	0.001	

When a diagnosis was later converted to bipolar disorder, mean (SD) time between the end of the first hospitalization period and the start of the hospitalization period including a diagnosis of bipolar disorder was 6.0(6.2) years, median 4.1 years.

Subsequent bipolar disorder diagnosis was most common among those first diagnosed with other severe mood (F32–39) and schizophrenia group (F20–29) disorders (Table 4).

**TABLE 4:** Diagnosis of bipolar disorder in subsequent hospitalization period according to diagnosis of the first hospitalization period. (%, *n*/*N*, Incidence rate per 100 follow‐up years).

	F00‐09, F70‐79, F80‐89, G	F10‐19, F60‐69, F90‐92	F20‐29	F32‐39	F40‐48, F50‐59, F93‐99	Z + Som	*p*
%	4.7	5.8	10.8	9.3	7.9	6.5	<0.001
*n*/*N*	13/277	111/1915	169/1566	236/2526	229/2892	29/448	
IR	0.33	0.42	0.70	0.92	0.55	0.35	

Multivariate analysis (Cox regression) showed that conversion to bipolar disorder diagnosis was more common among girls than boys. Conversion to bipolar disorder diagnosis was most common among adolescents treated for diagnosis of schizophrenia group (F20–29) and mood disorders (F30–39) in their index period. In multivariate analysis, conversion to bipolar disorder was similar across the decades (Table 5).

**TABLE 5:** Hazard Ratios (95% confidence intervals) for later diagnosis of bipolar disorder diagnosis among those with other diagnoses at their first admission at age 13–17 in three decades.

	HR (95% CI)	*p*
Sex		
Female	Ref	
Male	0.7 (0.6–0.8)	<0.001
Year of index admission		
2000–2010	Ref	
1980–1989	0.9 (0.7–1.1)	0.16
1999–1999	0.9 (0.8–1.1)	0.40
Primary diagnosis at index admission		
F00‐09, F70‐79, F80‐89, G	Ref	
F10‐19, F60‐69, F90‐92	1.2 (0.7–2.1)	0.56
F20‐29	2.1 (1.2–3.6)	0.01
F32‐39	2.0 (1.1–3.5)	0.01
F40‐48, F50‐59, F93‐99	1.5 (0.9–2.6)	0.15
Z group & somatic diagnoses	1.1 (0.6–2.2)	0.72

### 3.5 Mortality

The crude mortality rate among patients with bipolar disorder in the first treatment period (15/286) was the same (5.2%) as among those adolescents who were first hospitalized for other disorders (880/16826) (*p* 0.991). Mortality rate/1000 follow‐up years was 3.95 for bipolar disorder diagnoses and 3.86 for other diagnoses (*p* 0.93). No statistically significant differences in mortality rates were found between decades of first admission. The deaths were most commonly due to unnatural causes (suidice, violence, or accidents), with no difference between those with bipolar disorder at the index admission and those with other diagnoses (80.0% vs. 73.4%, *p* = 0.57).

## 4 DISCUSSION

In our nationwide data of first‐ever psychiatric inpatient treatment periods of adolescents aged 13–17, the absolute number of inpatient periods with a diagnosis of bipolar disorder (F30 or F31) increased decade by decade between 1980 and 2000 and was always greater among girls than boys. Incidence of inpatient‐treated bipolar disorder among 13‐ to 17‐year‐old adolescents was 2.8/100,000 over the whole study period, among same‐aged adolescents, and across decades treated incidence increased six‐fold. Those with a diagnosis of bipolar disorder during their first ever inpatient treatment were rehospitalized more often than those treated for other reasons. Less than half of bipolar diagnoses were retained in the patient's latest inpatient episode during follow‐up. On the other hand, only eight percent of other diagnoses converted to bipolar disorder. Mortality did not differ between those diagnosed with bipolar disorder during their first‐ever psychiatric inpatient episode and those treated for other reasons.

### 4.1 Treated incidence

Absolute numbers of hospital treated bipolar disorder and incidence per 100,000 same aged adolescents increased across the decades. Earlier studies have suggested that the incidence of bipolar disorder has increased significantly among adolescents and adults in the 21st century.^10^, ^11^, ^12^ Our findings corroborate this. However, the question remains whether this reflects true changes in morbidity among the population. True changes among adolescents could be due to, for example, the known secular trend of lowering of pubertal timing in Western countries, particularly among girls,^34^ followed by earlier onset of disorders mainly emerging in late adolescence or early adulthood. However, changes in diagnostic practices followed by increasing awareness of bipolar disorder cannot be ruled out.

Adolescent onset bipolar disorder in the present study was more common in girls than boys. The results concerning the difference between sexes have varied in earlier studies. Some studies have found no statistically significant difference in the incidence of bipolar disorder between sexes.^2^, ^8^ Other studies have found girls to be at greater risk of ending up in hospital due to bipolar disorder in adolescence and early adulthood, whereas boys were at greater risk in childhood.^12^

The proportion of bipolar diagnoses during first‐ever psychiatric hospitalization was greater in the group of middle adolescents (aged 15–17 years) than in that of early adolescents (aged 13–14 years) among both girls and boys. This concurs with the suggested first peak of incidence for bipolar disorder in adolescence/early adulthood (15–24 years).^2^

### 4.2 Rehospitalization

About two‐thirds of adolescents diagnosed with bipolar disorder were later rehospitalized. This exceeds the estimated proportion of recurrence of early‐onset bipolar disorder based on a systematic literature review.^6^ Rehospitalizations in our data may even not cover all recurrences since some episodes may be treatable without hospitalization. Therefore, the absolute rate of recurrences is likely still higher. These adolescents were also rehospitalized more often than those with other diagnoses at index admission. Rehospitalization likely suggests a severe course of the disorder. Higher rehospitalization rate of patients with bipolar disorder in Finland than elsewhere may suggest differences in diagnostic practices. Diagnostics perhaps focuses on severe type I bipolar disorders that end up more commonly rehospitalized and diagnosis of type II bipolar disorder diagnosis—less likely to require rehospitalization—may be used more conservatively than elsewhere.

### 4.3 Diagnostic stability and conversions

Less than half of bipolar diagnoses were retained in the patient's latest inpatient episode during follow‐up, a proportion lower than reported earlier based on a systematic review.^6^ On the other hand, only eight percent of other diagnoses converted to bipolar disorder. Concerns have been raised about delayed diagnosis of bipolar disorder and thus time lost before initiating the most appropriate treatments.^3^, ^4^, ^5^, ^6^ Our findings suggest that the opposite is also an issue: initial bipolar diagnosis may later require revision. Most of the diagnostic reclassifications, both from and to bipolar disorder, concerned bipolar disorder and other mood disorders (f32–39) or schizophrenia group diagnoses (f20–29), which concurs with the findings of earlier studies.^6^ The clinical picture of adolescent mania and schizophrenia may overlap, both possibly sharing symptoms of hallucinations, incoherence, and delusions.^5^ Overlapping symptoms and adolescent developmental phenomena (such as mood swings and lower behavioural controls) may complicate diagnostic work compared to diagnosing an adult with bipolar disorder, even if DSM‐5 field trials suggested good reliability of bipolar disorder diagnosis in both age groups.^35^ There is also great heterotypic continuity in mental disorders from childhood to adolescence to adulthood,^36^ i.e., morbidity persists but presentation changes across developmental phases.

Later conversion to bipolar diagnosis was rather rare. If conversion occurred, a diagnosis of bipolar disorder was set on average 6 years (median 4 years) after the first hospitalization at age 13–17 with some other diagnosis, which is in line with earlier findings.^15^, ^18^ Such delay need not relate to imprecise diagnostic work but may also be due to a prodromal phase with unspecific and changing symptoms.^37^, ^38^ Despite concerns about diagnosis of bipolar disorder being delayed, earlier research has to the best of our knowledge not focused on how commonly various other adolescent psychiatric disorders convert to bipolar disorder.

To summarize, the conditions fulfilling the diagnostic criteria for bipolar disorder and requiring inpatient care during adolescence in the present data appeared to be severe but their diagnostic stability was modest. Diagnostic uncertainty may be unavoidable due to unspecific prodromal phases and overlapping symptoms between severe mental disorders during adolescence and the known heterotypic continuity of mental disorders from childhood to adulthood.^39^ It is also possible that some differences between the current study and prior studies would be related to that diagnoses in the current study were based on hospital records, and there can have been variation in diagnostic procedures between hospitals and over time. Variation in diagnostic procedures has been suggested a key problem in research on pediatric bipolar disorder.^14^

### 4.4 Mortality

To the best of our knowledge earlier research has not focused on overall mortality among adolescents with bipolar disorder. Mortality among adolescents with bipolar disorder on their first‐ever admission did not differ from mortality among those initially treated for other disorders. Studies among adult patients likewise suggest that bipolar disorder increases the risk of mortality when compared to same aged general population, but not when compared to severe unipolar depression or schizophrenia.^20^, ^21^, ^22^, ^24^, ^26^

### 4.5 Methodological considerations

A strength of our study is a large nationwide register data. Even though the absolute number of inpatient treated bipolar disorder diagnoses was only 286, the data included all individuals admitted to psychiatric inpatient care for the first time in their lives at ages 13–17 during the period 1980–2010, were identified in the National Care Register for Health Care. The data were thus large and comprehensive. Reporting inpatient treatments to this register is mandatory. Also, data collection extending over three decades with a long follow‐up period allowed us to investigate possible trends. Extensive data from three decades with a long follow‐up period is one of the strengths of this study.

A possible limitation of our study could be the clinician‐dependent differences in diagnosing adolescent bipolar disorder. There may be variation between clinicians and across time in diagnostic work. Rigorously made structured research diagnoses would be more reliable, but not possible in this kind of a large register study. However, psychiatric diagnoses set in inpatient care in Finland have in general been shown to be very reliable,^40^, ^41^ also specifically concercing diagnosis of type I bipolar disorder.^42^, ^43^

Our study focused only on inpatient adolescent so the results cannot be generalized to general population or to all patients in outpatient care. Inpatient‐treated patients likely suffer from the most severe forms of psychiatric disorders. Their treatment needs are, however, likely the most complex, and this makes inpatient populations an important target of research.

## 5 CONCLUSION

The incidence of hospital‐treated adolescent onset bipolar disorder diagnoses increased from 1980 to 2010. Adolescents with bipolar disorder diagnoses were rehospitalized more often than those with other diagnoses. Diagnostic conversions occurred from and to unipolar depression and schizophrenia. This may be unavoidable, given the common heterotypic continuity of adolescent mental disorders. The findings do not suggest a considerable problem if delayed diagnosis of bipolar disorder among adolescents. Hospital‐treated adolescent bipolar disorder does not increase the risk of mortality when compared to other severe psychiatric disorders.

The variation in the diagnostic stability of bipolar disorder between decades points to service system‐related factors and to a need to increase uniform diagnostic practices. Increases in later reclassification of other diagnoses as bipolar disorder over the decades may also relate to external issues, such as increasing awareness and competencies regarding juvenile onset bipolar disorder.

## CONFLICT OF INTEREST STATEMENT

None.

## ETHICS STATEMENT

The study was duly approved by Tampere University Hospital ethics committee.

## Data Availability

The data that support the findings of this study are available on request from the corresponding author. The data are not publicly available due to privacy or ethical restrictions.
